# Congenital Hairy Polyp of the Palatopharyngeus Muscle

**DOI:** 10.1155/2013/374681

**Published:** 2013-06-19

**Authors:** Brandon Christianson, Seckin O. Ulualp, Korgun Koral, Dinesh Rakheja, Ronald Deskin

**Affiliations:** ^1^Department of Otolaryngology-Head and Neck Surgery, University of Texas Southwestern Medical Center and Children's Medical Center, Dallas, TX 75390, USA; ^2^Division of Pediatric Otolaryngology, University of Texas Southwestern Medical Center and Children's Medical Center, Dallas, TX 75390, USA; ^3^Department of Radiology, University of Texas Southwestern Medical Center and Children's Medical Center, Dallas, TX 75390, USA; ^4^Department of Pathology, University of Texas Southwestern Medical Center and Children's Medical Center, Dallas, TX 75390, USA; ^5^Department of Pediatrics, University of Texas Southwestern Medical Center and Children's Medical Center, Dallas, TX 75390, USA

## Abstract

*Purpose*. To describe clinical, radiologic, and histological features of a congenital hairy polyp arising from the palatopharyngeus muscle in a neonate. *Methods*. Chart of a 2-day-old female referred to a tertiary care pediatric hospital for assessment of intraoral mass was reviewed. *Results*. The child was born at 32 weeks and an intraoral mass was noted. The patient was transferred to tertiary care children's hospital on day 2 of life. The child had increased work of breathing at presentation and required continuous positive airway pressure. Physical examination revealed a pedunculated mass which was protruding into the oropharynx from the nasopharynx. MRI of the lesion documented a discrete bilobed mass which filled the posterior nasopharynx. The mass abutted the uvula and soft palate; however, the mass did not appear to be arising from the soft palate. Intraoperative exam showed a mass arising from the right palatopharyngeus muscle in the superior pole region of the tonsil. Histologic examination showed ectodermal and mesodermal derivatives confirming congenital hairy polyp. At 8-month followup, the surgical site was healed with no evidence of recurrent lesion. *Conclusions*. Congenital hairy polyp, though uncommon, should be considered in the differential diagnosis of oropharyngeal mass in neonates.

## 1. Introduction

Congenital hairy polyp is a rare malformation that consists of mesodermal and ectodermal elements [1]. Hairy polyp has shown 6 : 1 female predilection [1]. A wide variety of congenital anomalies including, but not limited to, cleft palate, agenesis of the uvula, external auricle, ankyloglossia, facial hemihypertrophy, left carotid artery atresia, and osteopetrosis have been documented in patients with hairy polyp [2].

To date, hairy polyp has been documented in the nasopharynx, soft and hard palate, tongue, pharynx, tonsil, palatopharyngeus and palatoglossus muscles, external auditory canal, middle ear, mastoid, hypopharynx, esophagus, and trachea [3]. This retrospective case review describes clinical, radiologic, and histological features of a congenital hairy polyp arising from the palatopharyngeus muscle in a neonate. 

## 2. Case Report

The child was born at 32 weeks to a mother with no medical problems. At the initial exam, an intraoral mass was noted and the patient was transferred to tertiary care children's hospital on day 2 of life. The child had increased work of breathing at presentation and required continuous positive airway pressure. Family history was unremarkable.

**Figure 1:**
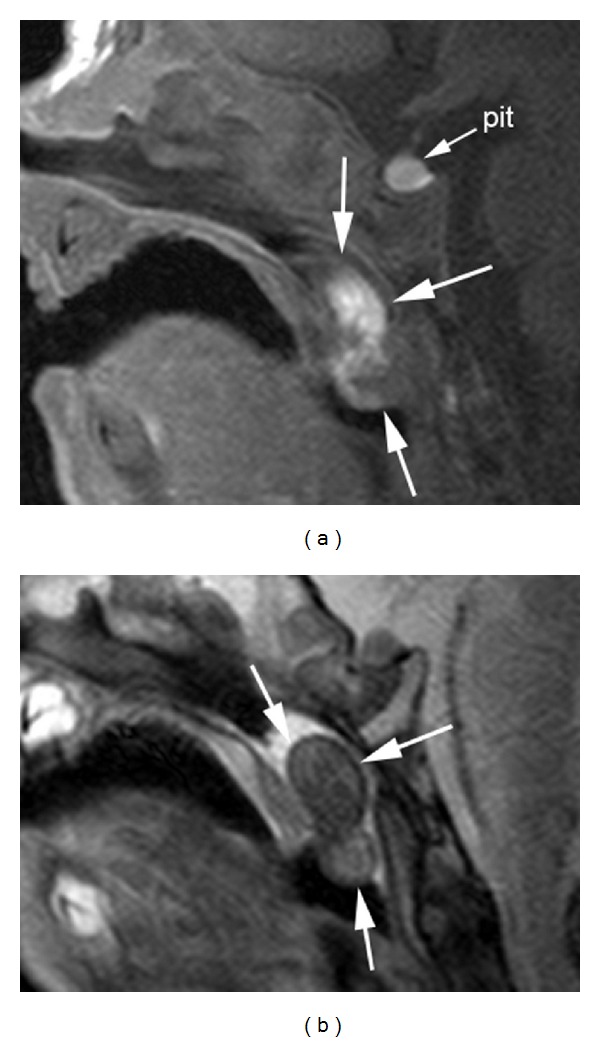
Sagittal short tau inversion recovery (STIR) (a) and sagittal T1-weighted (b) images of the nasopharynx show an oval, well-circumscribed mass (arrows) that is intimately related to the posterior aspect of the soft palate, but separate from it. The lesion is predominantly hyperintense on T1-weighted images and moderately and heterogeneously hyperintense on the T2-weighted images, features suggestive of fat content. Note the normal T1-weighted hyperintensity of the anterior pituitary (pit) in a newborn, presumably related to hypertrophy of the gland due to circulating maternal hormones.

**Figure 2:**
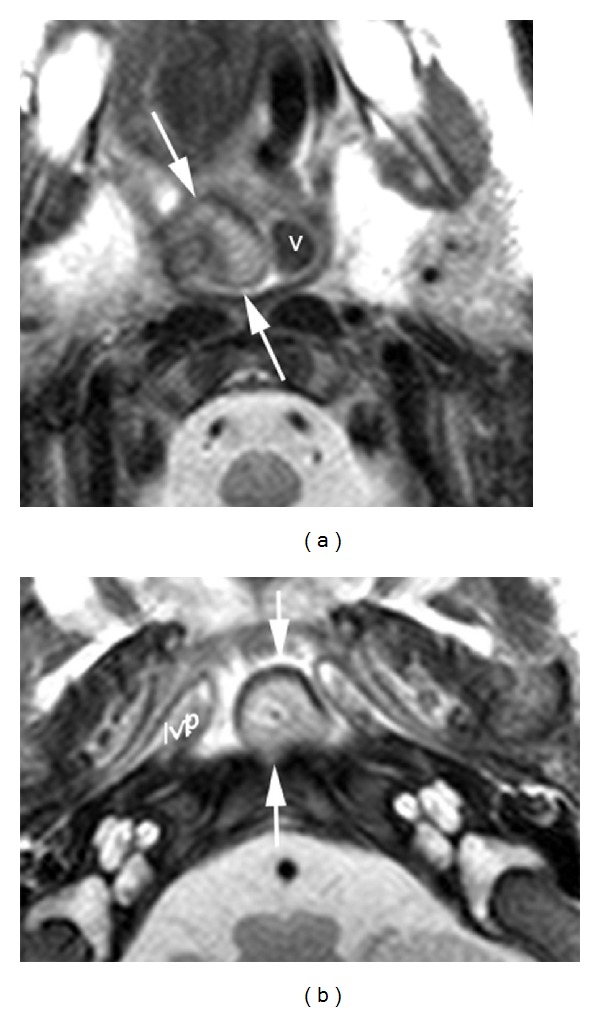
Axial T2-weighted image (a) at the level of the epiglottis displays that the lesion is arising from the right wall of the pharynx. The right vallecula is effaced. The left vallecula (v) is normal. Axial T2-weighted image (b) at the level of the nasopharynx shows the unattached margins of the mass (arrows). The black contour of the anterior wall of the lesion is due to chemical shift artifact, confirming the fat content of the lesion. Lvp: levator veli palatini muscle.

Physical examination revealed well-appearing child in no respiratory distress, normal otologic exam, and normal nasal exam. Oral cavity examination documented a pedunculated mass which was protruding into the oropharynx from the nasopharynx. No other abnormality was found in the head and neck region. Magnetic resonance imaging revealed a discrete mass of the posterior nasopharynx. The mass abutted the uvula and soft palate; however, the mass did not appear to be arising from the soft palate. The mass was predominantly hyperintense on T1-weighted images (Figure 1(a)) suggestive of fat content. On short tau inversion recovery (STIR) images, the mass was heterogeneous with regions of low and high signal intensity (Figure 1(b)). On T2-weighted images (Figures 2(a) and 2(b)), the mass was hyperintense and its relationship with the right side of the pharynx was better delineated. There was no restricted diffusion on the diffusion weighted images. 

**Figure 3:**
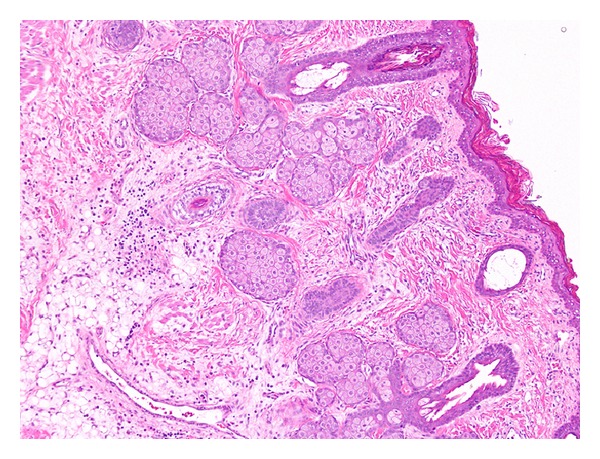
Photomicrograph of the lesion shows a lining of keratinizing stratified squamous epithelium with underlying pilosebaceous units embedded in fibrous tissue. The deeper portion of the lesion shows fibroadipose tissue and focal groups of skeletal muscle fibers (hematoxylin and eosin, 100x magnification).

Intraoperative exam showed a mass arising from the right palatopharyngeus muscle in the superior pole region of the tonsil. The lesion was removed using monopolar diathermy under general anesthesia. Histological evaluation showed a polypoidal lesion lined by keratinizing stratified squamous epithelium (Figure 3). Subjacent to the epithelium was a zone of fibrous connective tissue, deep to which was variably mature adipose tissue divided into lobules by fibrous septa. Many sebaceous glands, eccrine glands, and hair follicles were embedded in the fibrous/fibroadipose tissue. Many skeletal muscle fibers were present focally, arranged perpendicular to the surface.

Postoperatively, respiratory difficulties were completely resolved. The surgical site was healed with no evidence of recurrent lesion at 8-month followup.

## 3. Discussion

Hairy polyps are rare benign tumors usually appearing during the early days of life. The most common localization for hairy polyp is the nasopharynx, usually originating from the soft palate or lateral pharyngeal wall. To date, hairy polyp of the palatopharyngeus muscle has been reported in two neonates [4, 5]. Previous studies have documented clinical and histologic features of hairy polyp of the palatopharyngeus muscle [4, 5]. Here we described radiologic features of hairy polyp of the palatopharyngeus muscle in addition to clinical presentation and histological characteristics.

Hairy polyp usually presents as a sausage or pear-shaped, pedunculated mass with limited growth potential [3]. Malignant transformation has not been reported. Depending on the size and location of the mass, the clinical presentations may include a wide variety of symptoms such as respiratory distress, stridor, cyanosis, snoring, feeding difficulty, failure to gain weight, drooling, hemoptysis, and coughing [3]. In the presence of airway obstruction, airway must be secured. Enteral nutritional support should be considered when feeding difficulty, dysphagia, or failure to thrive occurs. The clinical differential diagnoses for naso/oropharyngeal mass in children encompass a wide spectrum of lesions including, but not limited to, teratoma, encephalocele, craniopharyngiomas, chordomas, hamartomas, gliomas, neurofibromas, rhabdomyosarcomas, and vascular anomalies [4]. 

Radiologic assessment of a naso/oropharyngeal mass in a neonate is useful to assess the origin and extent of the mass, to aid differential diagnosis, to determine an intracranial extension, and to plan surgery. Computed tomography provides assessment of the mass and the surrounding bony changes; however, radiation exposure is a concern. Magnetic resonance imaging (MRI) aids diagnosis and surgery planning by delineating the characteristics and extent of the mass and its relationship to the vascular and muscular structure. Imaging characteristics of hairy polyp include a polypoid lesion consisting mainly of lipid with a usually fibrous stalk and no intracranial or intraspinal extension [6]. High signal intensity on T1-weighted images is caused by the high fat content of the hairy polyp. The high fat content narrows the differential diagnosis of a neonatal naso/oropharyngeal mass to a hamartoma, teratoma, or dermoid and is useful to exclude lesions such as neuroblastoma, meningoencephaloceles, vascular anomalies, and embryonic cysts [6–8]. In our case, the mass was predominantly hyperintense on T1-weighted images supporting the high fat content of the hairy polyp. Diffusion weighted MR imaging revealed no restricted diffusion and ruled out the possibility of dermoid, which shows hyperintensity on the diffusion weighted images.

Histological examination is crucial in differentiating hairy polyp from other lesions such as teratomas, hamartomas, dermoid cyst, and choristoma. Hairy polyp is composed of tissues derived from mesoderm and ectoderm. Mesoderm derived tissues may include fibroadipose tissue, muscle, or cartilage. Ectodermal component includes keratinizing stratified squamous epithelium and skin appendages. Unlike hairy polyp, teratomas consist of tissues derived from all three germinal cell layers. Hamartomas differ from hairy polyp as hamartomas are characterized by excessive or haphazardly formed but histologically normal tissue for the anatomical location. Dermoid cysts contain desquamated epithelium and keratinous debris. A choristoma is a mass composed of histologically normal tissue in an anatomically abnormal location. The treatment of choice for hairy polyp is surgical excision and definitive diagnosis is made by histologic examination. No recurrence has been reported after complete excision. 

In conclusion, congenital hairy polyp, though uncommon, should be considered in the differential diagnosis of oropharyngeal mass in neonates. Otolaryngologists, radiologists, and pathologists should be aware of the occurrence of congenital hairy polyp in the pediatric age group.
